# Hygiene and Public Health

**Published:** 1905-12

**Authors:** 


HYGIENE AND PUBLIC HEALTH. By B. Arthur Whitelegge, C. B., M.D., B.Sc. Lond., F.R.C.P., D.P.H. Late County Medical Officer of Health for the West Riding of Yorkshire, Chief Sanitary Officer of the West Riding of Yorkshire Rivers Board, Medical Officer of Health for Nottingham, etc. etc., and George Newnan, M.D., D.P.H., F.R.S.C., Medical Officer of Health of the Metropolitan Borough of Finsbury, Late Demonstrator of Bacteriology and Infective Diseases in King's College, London, etc. New edition, revised enlarged, and in great part rewritten. Illustrated. Price $1.75 net. W. T. Keener & Co., Chicago, Ill.

The subject of hygiene and public health comprises so many branches of medicine as epidemology, bacteriology, chemistry, physics, meteorology, geology, engineering, etc., that no one book could contain everything. This manual, though, has for its object to summarize the important points in all these subjects and present them in a way that makes them of practical value in preventive medicine and to medical officers of health. Rapid and important strides are to be taken along this line of preventive medicine, and this book will prove of value to any one desiring the important features in a brief form.

The only criticism we desire to make is that *venereal diseases* and their fountain-head *prostitution* are entirely ignored. If there is any factor making a more vigorous attack on public health, or one demanding more attention in all nations to-day than these questions we are not cognizant of them.

